# Tourniquet application in primary total knee arthroplasty for osteoarthritis: A systematic review and meta-analysis of randomized controlled trials

**DOI:** 10.3389/fsurg.2022.994795

**Published:** 2023-01-06

**Authors:** Jinchang Han, Xiao-yu Zhang, Shi-yin Mu, Shi-long Liu, Qing-tong Cui, Chao Zhang, Ai-feng Liu

**Affiliations:** ^1^ Department of Orthopedics, First Teaching Hospital of Tianjin University of Traditional Chinese Medicine, Tianjin, China; ^2^ National Clinical Research Center for Chinese Medicine Acupuncture and Moxibustion, Tianjin, China; ^3^ Department of Respiratory of Machang, Tianjin Children's Hospital/Tianjin University Children's Hospital, Tianjin, China

**Keywords:** total knee arthroplasty, tourniquet, blood loss, meta-analysis, transfusion requirement

## Abstract

**Objective:**

The aim of this study was to identify the influence of a tourniquet on the blood loss, transfusion requirement, swelling, pain, knee function, range of motion (ROM), operation time, bone cement mantle thickness, and complications in patients operated with total knee arthroplasty (TKA).

**Methods:**

Two authors independently retrieved PubMed, Embase, and CENTRAL to identify eligible randomized controlled trials (RCTs) evaluating the effectiveness of a tourniquet in TKA. Fixed- (*I*^2 ^< 50%) or random-effects (*I*^2 ^> 50%) models were selected to perform meta-analysis according to the value of *I*^2^. Mean difference (MD) and risk ratio were selected as the effect sizes for continuous and dichotomous variables, respectively.

**Results:**

A total of 29 RCTs, involving 2,512 operations (1,258 procedures with a tourniquet and 1,254 procedures without a tourniquet), were included, and 18 outcomes were compared. Tourniquet application could significantly decrease intraoperative blood loss (MD = −138.72 ml, *p* < 0.001), shorten operation duration (MD = −1.77 min, *p* < 0.001), and increase cement mantle thickness (MD = 0.17 mm, *p* < 0.001). However, it was significantly associated with increased postoperative pain intensity, decreased full ROM/flexion ROM/extension ROM, poorer knee function, increased knee swelling, and increased length of hospital stay (LOS) at several follow-up points (*p* < 0.050). No significant difference was found for postoperative draining volume, total blood loss, transfusion rate, change of Hb level, and risks of deep venous thrombosis and all complications.

**Conclusions:**

Tourniquet application could only decrease the intraoperative blood loss but has no effectiveness on the total blood loss and transfusion requirement. On the contrary, it has a reverse effect on the pain score, knee function, ROM, swelling, and LOS.

## Introduction

Total knee arthroplasty (TKA) has been shown to be with a satisfaction rate of up to 91%, for patients with end-stage knee osteoarthritis (1, 2). A successful TKA procedure depends on multiple critical factors, among which the bloodless operation field is an important factor to guarantee a full visualization during operation, decrease blood loss and requirement of blood transfusion, and reduce operation time (3, 4). Various strategies have been deployed to reduce intraoperative blood loss in TKA, including iron therapy, administration of erythropoietin, controlled hypotension, local or systemic administration of tranexamic acid (TXA), and application of a tourniquet (4–6). The pneumatic tourniquet was first applied in surgery as described by Cushing et al. (3) in 1904. In the recent publications, over half of surgeons prefer to use a tourniquet during TKA (7, 8).

However, although the tourniquet is widely accepted by many orthopedic surgeons, results from clinical trials remain controversial (4, 9–12). There are a growing body of studies that have raised some concerns about the tourniquet application during TKA, mainly for the increased risks of postoperative pain, reperfusion injury, limb swelling, deep venous thrombosis (DVT), wound complication, and peripheral nerve injury (9–11). In a meta-analysis conducted by Cai et al. (4) in 2019, 11 primary randomized controlled trials (RCTs) involving 541 patients were included for analysis, showing that tourniquet application during TKA could only decrease the intraoperative blood loss and calculated blood loss, but has no effectiveness on postoperative blood loss, transfusion rate, total blood loss, and risk of DVT. Smith and Hing (12) conducted a meta-analysis based on 15 RCTs or non-RCTs, presenting that tourniquet-assisted TKA was associated with a significantly increased intraoperative blood loss compared to the non-tourniquet-assisted group. No significance was found for the total blood loss and overall transfusion rate. However, the application of a tourniquet was related with a trend of increased complications. In recent years, plenty of new studies have been published to discuss the effectiveness of tourniquet application in TKA, but no consensus has been reached until now.

Thus, in response to this confusion about the use of a tourniquet, this meta-analysis was aimed to identify the influence of a tourniquet on the blood loss, transfusion requirement, swelling, pain, knee function, range of motion (ROM), operation time, bone cement mantle thickness, and complications in patients operated with TKA. The working hypothesis of this review is that the application of a tourniquet could reduce blood loss and transfusion requirement but can have adverse effects on swelling, pain, knee function, ROM, operation time, bone cement mantle thickness, and complications in primary TKA.

## Materials and methods

### Study search

The current review was conducted according to the PRISMA statement (13). Two authors searched the electronic databases of PubMed, Embase, and Cochrane Library independently to identify potentially related records. The key words include “total knee arthroplasty” and “tourniquet.” We conducted the search on the platforms using a method of combining the “subject term” and “free terms.” There was no restriction on the publication years (from inception of the databases to year of 2021) and publication countries.

### Inclusion and exclusion criteria

The records retrieved from the electronic databases were screened for eligibility when fulfilling the following criteria: (1) participants: undergone TKA for knee osteoarthritis; (2) intervention and comparison: the TKA was assisted with or without a tourniquet during operation; (3) outcome: studies reported outcome data about blood loss, transfusion requirement, swelling, pain, knee function, ROM, operation time, bone cement mantle thickness, complications, and so on; (4) study: only RCTs with high quality were included.

Studies were excluded for the following reasons: (1) duplicated records; (2) studies published in non-English language; (3) studies for patients with diagnosis other than knee osteoarthritis; (4) non-RCTs, including cohort study, nonrandomized clinical trial, case–control study, case series, literature review, and meta-analysis.

Two authors independently screened the records to identify eligible studies. First, all available records were checked to remove the duplicates. The remaining records were then evaluated for potential eligibility by screening the titles/abstracts and the full text, in turn.

### Data extraction

Two reviewers extracted the related information independently. In case of diversity on the extracted data between two reviewers, a third senior author would participate in the discussion. The following data were extracted: (1) Study information: first author's name, year of publication, publication country, and study period; (2) Patients’ information: sample size, number of dropped patients, sex, average age, body mass index (BMI), and follow-up time; (3) Intervention information: tourniquet product, tourniquet pressure applied, prostheses types, type and volume of cement, postoperative thromboprophylaxis, and other management of intraoperative bleeding; (4) Clinical outcomes: data about the operation time, intraoperative blood loss, draining volume, total blood loss, change of hemoglobin (Hb) level, transfusion requirement, length of hospital stay (LOS), bone cement mantle thickness, visual analog scale (VAS)-pain score, ROM, Knee Society Score (KSS), Oxford knee score, knee swelling, and risks of DVT and all recorded complications.

The risk of bias of primary RCTs was evaluated using the Cochrane Collaboration tool (14), by two individual authors.

### Statistical analysis

For those variables reported as mean ± standard deviation (SD), the mean difference (MD) as well as 95% confidence interval (95% CI) were selected as the effect size, while for those outcomes reported as percentage or dichotomous variable, risk ratio (RR) was selected as effect size. Random- (*I*^2 ^> 50%) or fixed-effects (*I*^2 ^< 50%) models would be selected according to the between-study heterogeneity test.

For the continuous variables, subgroup meta-analyses were performed at different follow-up points and were displayed in line charts. In each meta-analysis with significant heterogeneity, if three or more studies were included, sensitivity analysis was conducted to detect studies causing unstability. Publication bias tests, including Begg's test (*p* < 0.050) and Egger's test (*p* < 0.100), were performed, when five or more studies were included (15). When significant publication bias was detected, a nonparametric trim-and-fill method would be used to adjust the bias.

The above procedures were performed with R version 4.0.5 (R Foundation for Statistical Computing, Vienna, Austria). A two-sided *p* value of <0.05 was defined as statistical significance.

## Results

### Study searching and screening

**Figure 1:**
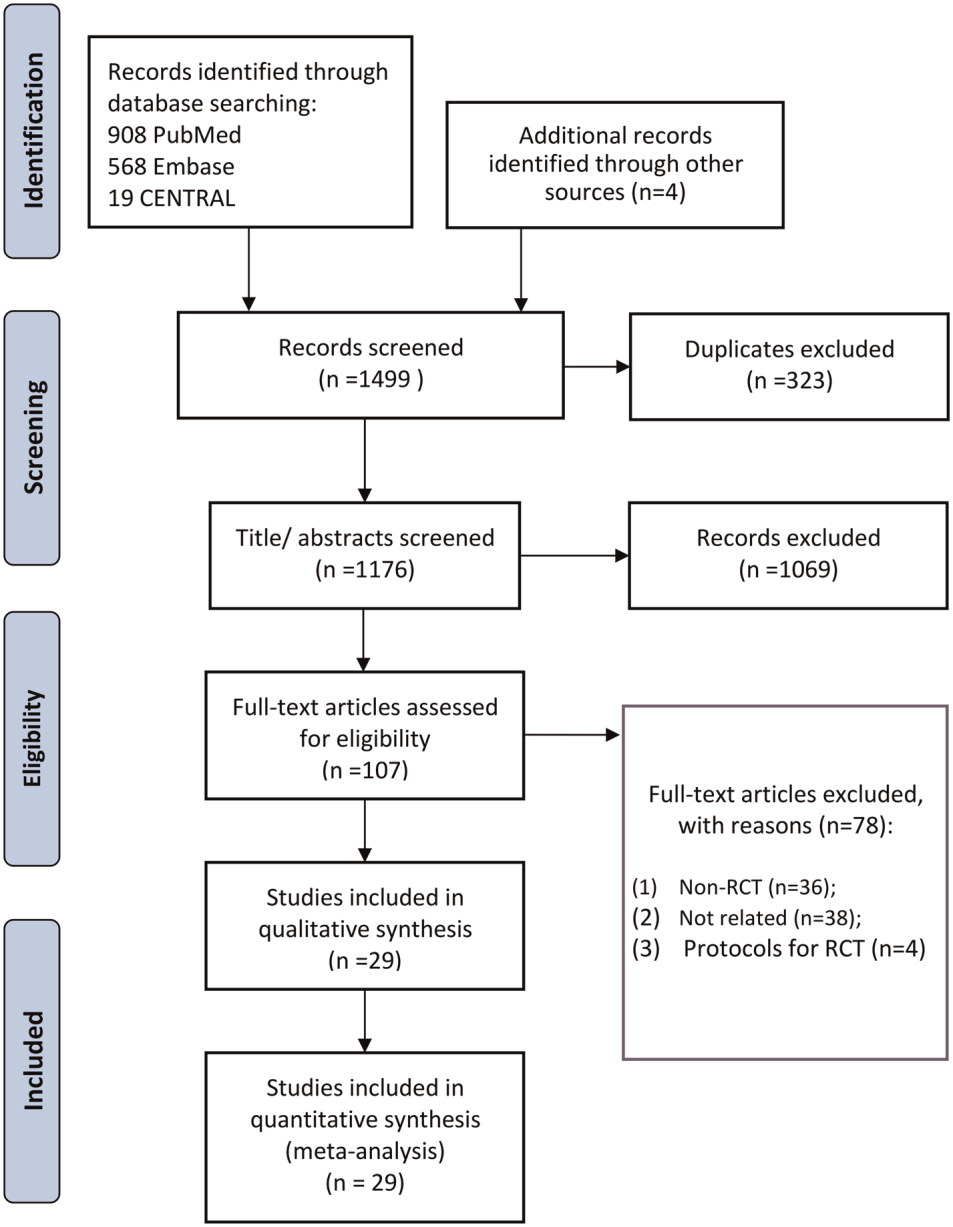
Flowchart of study searching and selecting.

The search and screening flowchart is presented in Figure 1. First, 1,499 records were identified through database retrieval and manual search. After excluding 323 duplicates, 1,069 titles/abstracts were excluded. The remaining 107 were assessed by reading the full text, 78 of which were excluded with various reasons. Finally, a total of 29 RCTs (9, 16–42) were eligible for final inclusion in qualitative synthesis and quantitative meta-analysis.

### Basic information of the included trials

Table 1 presents the summary of the included studies. A total of 2,512 operations (2,393 patients, 119 patients conducted bilateral TKA) were included. With a tourniquet, 1,258 procedures were performed and 1,254 were performed without a tourniquet. The male percentages ranged from 14% to 70% in the tourniquet group and from 10.3% to 90% in the non-tourniquet group. The mean age at operation was available in all studies, ranging from 58.0 to 73.0 both in tourniquet and non-tourniquet groups. The BMI was available in 21 studies, ranging from 26.1 to 33.0 in the tourniquet group and from 25.3 to 33.0 in the non-tourniquet group. At the final follow-up, a total of 45 and 49 procedures were dropped out in the tourniquet and non-tourniquet groups, respectively.

**Table 1:** Characteristics of the included trials.

Author	Year	Country	Study period	Group	Number	Male%	Age	BMI	Drop out	Follow-up
Yi (16)	2021	China	May 2017–Jun 2018	TQT	50	14	68.4 ± 6.8	26.1 ± 2.6	0	3 months
				Non-TQT	50	16	68.0 ± 7.1	25.3 ± 3.6	0	
Wall (17)	2021	United Kingdom	Apr 2017–Feb 2018	TQT	27	59	69.4 ± 6.9	30.5 ± 5.5	2	12 months
				Non-TQT	26	50	67.5 ± 6.8	30.6 ± 5.2	3	
Zhao (18)	2020	China	Jan 2017–Dec 2018	TQT	60	62	65.0 ± 9.6	26.4 ± 3.5	0	3 months
				Non-TQT	60	67	64.5 ± 8.54	25.7 ± 5.4	0	
Palanne (19)	2020	Finland	Oct 2016–Dec 2018	TQT	204	33	64.0 ± 7.0	30.6 ± 4.4	4	1 day
				Non-TQT	200	40	64.0 ± 7.0	30.2 ± 4.3	5	
Ayik (20)	2019	Turkey	Feb 2015–Nov 2017	TQT	35	43.8	65.4 ± 7.3	31.4 ± 4.7	3	3 months
				Non-TQT	35	42.4	64.9 ± 6.6	30.3 ± 7.1	2	
Ajnin (21)	2019	United Kingdom	Jan 2014–Dec 2016	TQT	29	N/A	73 (range: 58–81)	33.0 (range: 28–42)	0	8 months
				Non-TQT	29					
Goel R (22)	2019	United States	Jun 2016–Apr 2017	TQT	100	50	66.0 ± 7.0	30.9 ± 4.6	16	8 months
				Non-TQT	100	48	65.5 ± 7.8	31.3 ± 4.5	18	
Jawhar (23)	2019	Germany	NA	TQT	50	34	69.3 ± 7.4	31.9 ± 6	5	6 months
				Non-TQT	49	38.8	68.3 ± 7.8	31.4 ± 5.5	2	
Jawhar (24)	2018	Germany	NA	TQT	43	37.2	70.0 ± 6.8	31.9 ± 5.7	0	6 days
				Non-TQT	43	37.2	71.0 ± 6.8	31.9 ± 5.7	0	
Alexandersson (25)	2018	Sweden	Sept 2012–Jun 2015	TQT	38	47.4	68.0 ± 7.4	28.6 ± 3.4	1	3 months
				Non-TQT	43	51.2	69.7 ± 6.4	27.9 ± 3.5	3	
Ozkunt (26)	2018	Turkey	Jan 2015–Jan 2016	TQT	24	N/A	65.1 (range: 52–81)	N/A	0	6 weeks
				Non-TQT	25				0	
Zhou (27)	2017	China	Jan 2016–May 2016	TQT	74	18.1	66.8 ± 8.6	26.1 ± 4.1	2	6 months
				Non-TQT	74	10.3	69.1 ± 7.6	25.7 ± 3.4	6	
Liu (28)	2017	China	Jan 2010–Jan 2015	TQT	52 knees	30.8	67.0 ± 8.0	28.1 ± 5.5	4	25 (range: 19–36) months
				Non-TQT	52 knees	30.8	67.0 ± 8.0	28.1 ± 5.5	4	
Vertullo (29)	2017	Australia	NA	TQT	20	50	67.9 ± 6.9	30.4 ± 5.1	0	N/A
				Non-TQT	20	55	65.7 ± 8.5	31.0 ± 5.3	0	
Dennis (9)	2015	United States	Oct 2012–Aug 2014	TQT	28 knees	57.1	62.0 ± 6.0	29.0 ± 4.0	0	3 months
				Non-TQT	28 knees	57.1	62.0 ± 6.0	29.0 ± 4.0	0	
Kumar (30)	2015	India	Jul 2013–Jan 2014	TQT	30 knees	30	58.0 (range: 45–69)	N/A	0	6 weeks
				Non-TQT	30 knees	30			0	
Harsten (15)	2015	Sweden	Aug 2013–May 2014	TQT	32	53.1	68.0 ± 8.0	N/A	0	2 days
				Non-TQT	32	56.3	66.0 ± 8.0		0	
Liu (31)	2014	Australia	NA	TQT	10	70	67.0	N/A	0	12 months
				Non-TQT	10	90	70.0		0	
Pfitzner (32)	2014	Germany	NA	TQT	45	46.7	69.3 (range: 47–85)	27.8 (range: 18.5–38.1)	0	2 weeks
				Non-TQT	45	24.4	70.5 (range: 50–90)	26.0 (range: 18.5–33.9)	0	
Molt (33)	2014	Sweden	Sept 2008–Jun 2009	TQT	30	53.3	70.0 ± 7.0	28.0 ± 3.0	8	2 years
				Non-TQT	30	53.3	67.0 ± 9.0	28.0 ± 3.0	4	
Tai (34)	2012	China	NA	TQT	36	25	72.1 ± 6.9	28.6 ± 4.5	0	4 days
				Non-TQT	36	22.2	71.5 ± 6.8	27.9 ± 4.2	0	
Ledin (35)	2012	Sweden	Jun 2007–Apr 2009	TQT	25	40	70.0 ± 8.0	29.0 ± 4.8	0	2 years
				Non-TQT	25	39.1	71.0 ± 6.0	28.0 ± 4.8	2	
Yavarikia (36)	2009	Iran	2005–2008	TQT	22 (29 knees)	27.3	68.0 (range: 4–72)	N/A	0	2 days
				Non-TQT	29 (31 knees)	24.1	66.0 (range: 51–74)		0	
Li (37)	2008	China	Aug 2007–Apr 2008	TQT	40	27.5	71.0 ± 6.0	27.3 ± 6.3	0	2 weeks
				Non-TQT	40	32.5	70.0 ± 7.0	26.8 ± 5.1	0	
Fukuda (38)	2007	Japan	Apr 2003–Apr 2005	TQT	27	14.8	71.2 ± 8.2	26.1 ± 3	0	5 days
				Non-TQT	21	14.3	73.1 ± 5.6	26.5 ± 2.8	0	
Vandenbussche (39)	2002	France	Dec 1997–Dec 1999	TQT	40	22.5	72.5 (range: 38–89)	N/A	0	3 months
				Non-TQT	40	40	68.5 (range: 50–81)		0	
Tetro (40)	2001	Canada	N/A	TQT	33	45.5	69.8 ± 6.7	N/A	0	1 week
				Non-TQT	30	36.67	69.8 ± 9.0		0	
Aglietti (41)	2000	Italy	N/A	TQT	10	30	70.0 ± 8.0	27.9	0	1 day
				Non-TQT	10	40	68.0 ± 4.5	27.3	0	
Wakankar (42)	1999	United Kingdom	N/A	TQT	37	29.7	72.5 (range: 57–85)	N/A	0	4 months
				Non-TQT	40	35	71.8 (range: 43–91)		0	

N/A, not applicable; TQT, tourniquet group; non-TQT, non-tourniquet group; BMI, body mass index.

Table 2 provides a list of detailed information about the operation, including tourniquet product, tourniquet pressure, prosthesis and bone cement used for TKA, postoperative thromboprophylaxis, and other management of intraoperative bleeding. The tourniquet pressure was reported in 24 trials. In general, they applied a higher pressure of 100–150 mmHg than systolic blood pressure (SBP) or an absolute pressure of 250–380 mmHg.

**Table 2:** Detailed information about the operation procedure.

Study ID	Tourniquet product	Tourniquet pressure	Prostheses used for TKA	Bone cement used for TKA	Thromboprophylaxis	Other management of intraoperative bleeding
Yi (16)	VBM, Germany	100 mmHg + SBP	Posterior-stabilized fixed bearing P.F.C TKA (DePuy, Warsaw, IN, United States)	Smartest GMV Endurance, DePuy, Blackpool, England	0.3 ml (3,000 IU) LMWH	3 g of i.v. TXA and 1 g topical TXA
Wall (17)	N/A	N/A	N/A	N/A	(1) IPCC; (2) LMWH	N/A
Zhao (18)	N/A	100 mmHg + SBP	Genesis II Total Knee System; Smith & Nephew, Memphis, TN, United States	N/A	N/A	i.v. TXA (20 mg/kg)
Palanne (19)	N/A	250 mmHg	Cemented Triathlon® Total Knee System (Stryker, Kalamazoo, MI, United States)	N/A	N/A	i.v. TXA 1 g and ondansetron 4 mg
Ayik (20)	Tourniquet 2,500 ELC, VBM, Sulzam Neckar, Germany	100 mmHg + SBP	GENESIS II cemented, posterior cruciate ligament-retaining, fixed bearing total knee endoprosthesis	Ultra-high-molecular weight polyethylene (Smith & Nephew Orthopedics, Inc., Memphis, TN, United States)	LMWH	None
Ajnin (21)	N/A	300 mmHg	Cruciate-retaining prosthesis (Triathalon, Stryker)	Bone cement with Gentamycin	Enoxaparin 40 mg subcutaneously	i.v. TXA 1g
Goel (22)	The Stryker Color Cuff Dual Port	300/225 mmHg depending on preference	The DePuy Synthes P.F.C. SIGMA or Zimmer Biomet Persona implant system	N/A	Aspirin, 81 mg twice a day	i.v. TXA 1g
Jawhar (23)	Balbina™, Ulrich Medical, Ulm, Germany	360 mmHg	PFC® SIGMA®, DePuySynthes, Warsaw, IN, United States	SmartSet Bone cement, (DePuySynthes, Warsaw, IN, United States)	N/A	None
Jawhar (24)	Pneumatic tourniquet (Balbina™, Ulrich Medical, Ulm, Germany)	360 ± 20 mmHg	Cemented PFC® SIGMA® prosthesis (DePuySynthes, Warsaw, IN, United States)	40 g (SmartSet Bone cement, DePuySynthes, Warsaw, IN, United States)	N/A	N/A
Alexandersson (25)	Tourniquet (34 in., single bladder, dual port, Zimmer)	300 mmHg	Cemented NexGen CR- or PS-Flex fixed bearing knee (Zimmer) prosthesis	N/A	LMWH (Fragmin, 5,000 IE subcutaneously)	i.v. TXA 1 g
Ozkunt (26)	N/A	N/A	Posterior cruciate-retaining Genesis II (Smith & Nephew, Memphis, TN, United States) cemented knee system	OrCem 3 low viscosity PMMA (European Medical Contract Manufacturing, Nijmegen, Netherlands)	LMWH (4,000 IU)	N/A
Zhou (27)	N/A	N/A	Sigma fixed or rotating plant posterior-stabilized total knee prosthesis (PFC, Johnson & Johnson/DePuy, Warsaw, IN, United States)	N/A	Oral rivaroxaban (10 mg/day)	i.v. TXA
Liu (28)	N/A	125 mmHg + SBP	Posterior-stabilized knee prostheses (26 GENESIS II [Smith & Nephew, Memphis, TN, United States]	N/A	Oral rivaroxaban (10 mg/day)	N/A
Vertullo (29)	N/A	300 mmHg	NexGen posterior-stabilized LPS Mobile Bearing knees (Zimmer, Warsaw, IN, United States)	Mixing using 80 g of vacuum mixed Palacos RþG PMMA (Zimmer)	N/A	N/A
Dennis (9)	N/A	250 mmHg	N/A	N/A	N/A	N/A
Kumar (30)	N/A	100 mmHg + SBP	N/A	N/A	N/A	N/A
Harsten (15)	Tourniquet 2,500 (VMB Medizin Tecknik, Germany)	100 mmHg + SBP	Triathlon™ Knee System (Stryker, Mahwah, NJ, United States)	N/A	N/A	i.v. TXA 1g
Liu (31)	VBM Medizintechnik GmbH, Sulz, Germany	300 mmHg	Cemented fixed bearing posterior cruciate-retaining prosthesis	N/A	N/A	N/A
Pfitzner (32)	N/A	350 mmHg	Cemented, posterior-stabilized primary TKA (Nexgen LPS Flex, Zimmer, Warsaw, IN, United States)	40 g (Palacos R®, Heraeus, Hanau, Germany)	N/A	N/A
Molt (33)	N/A	300 mmHg	Cemented Triathlon™ (Stryker, Mahwah, NJ, United States)	Refobacin® Bone Cement R (Biomet Inc., Warsaw, IN, United States)	LMWH	i.v. TXA
Tai (34)	N/A	100 mmHg + SBP	Genesis II Total Knee System (Smith & Nephew, Memphis, TN, United States) or U2 Knee System (United Orthopedic, Taipei, Taiwan)	N/A	None	N/A
Ledin (35)	Standard tourniquet (Stille AB, Solna, Sweden)	275 mmHg	Nexgen CR all-poly tibia knee prosthesis (Zimmer)	Palacos R + G (Heraeus Medical Nordic, Sollentuna, Sweden) (40 g Palacos and 0.5 g gentamicin)	LMWH (Innohep, 4,500 IU subcutaneously)	N/A
Yavarikia (36)	N/A	380 mmHg	Scorpio (Stryker, United States) and Nexgen (Zimmer, United States)	N/A	LMWH (Enoxaprine)	N/A
Li (37)	N/A	100 mmHg + SBP	Genesis II, Smith & Nephew Inc., Memphis, TN, United States	N/A	(1) IPCC; (2) LMWH; (3) Early mobilization	3 g of hemostatic powder (Arista, Medafor, Minneapolis, MN, United States)
Fukuda (38)	N/A	350 mmHg	N/A	N/A	N/A	Foot pump (A-V Impulse System; Novamedix, Andover, United Kingdom)
Vandenbussche (39)	N/A	350 mmHg	Wallaby I (Protek-Sulzer, Bern, Switzerland)	Palacos cement with gentamicin (Palacos-Genta, Schering-Plough)	N/A	Enoxaparine
Tetro (40)	N/A	125∼150 mmHg + SBP	Johnson & Johnson, New Brunswick, NJ, United States; /Omnifit 7,000 prosthesis (Osteonics, Allendale, NJ, United States); /AMK components (Depuy Orthopedics, Warsaw, IN, United States)	N/A	Coumadin	N/A
Aglietti (41)	N/A	N/A	M.B.K prosthesis, Zimmer, Warsaw, IN, United States	N/A	N/A	N/A
Wakankar (42)	N/A	N/A	Insall-Burstein Mark II (Zimmer Ltd, Swindon, United Kingdom)	N/A	Low-dose warfarin	N/A

N/A, not available; TKA, total knee arthroplasty; LMWH, low-molecular weight heparin; SBP, systolic blood pressure; IPCC, intermittent pneumatic calf compression; TXA, tranexamic acid; i.v., intravenous.

**Figure 2:**
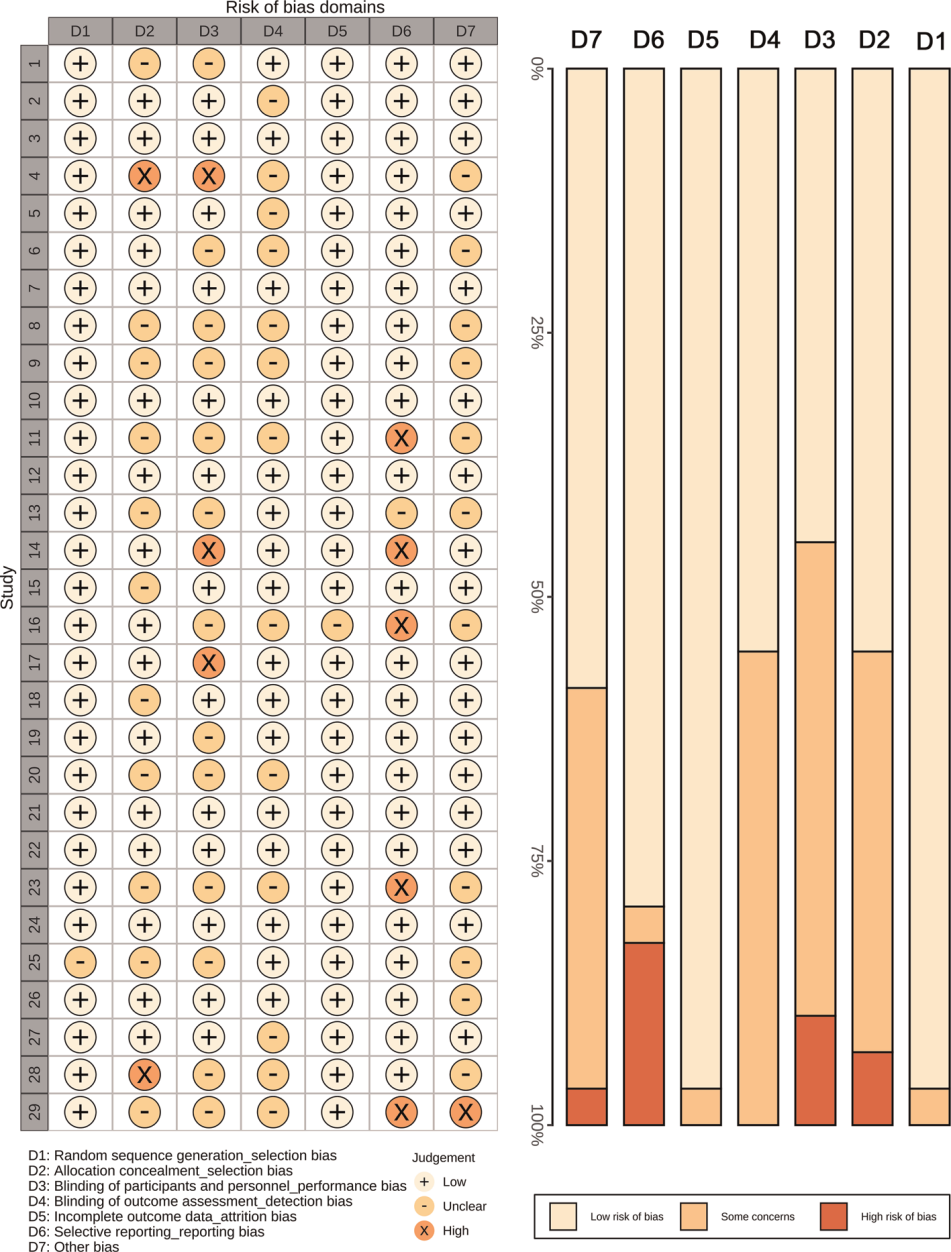
The risk of bias summary plot and bar chart for risk of bias assessment. A total of seven domains were assessed. A few studies have high risk of bias concerning the allocation concealment, blinding of participants, selective report, and other bias.

Figure 2 shows the results of assessment on the risk of bias. Generally, no obvious risk of bias was demonstrated in these trials. Only a few studies have high risk of bias, on the domains 2 (allocation concealment), 3 (blinding of participants), and 6 (selective reporting).

### Results of meta-analyses

A total of 18 outcomes were compared in this study, and the results of meta-analyses as well as the additional analyses (heterogeneity analysis, sensitivity analysis, and publication bias test) are presented in Supplementary Table S1.

**Figure 3:**
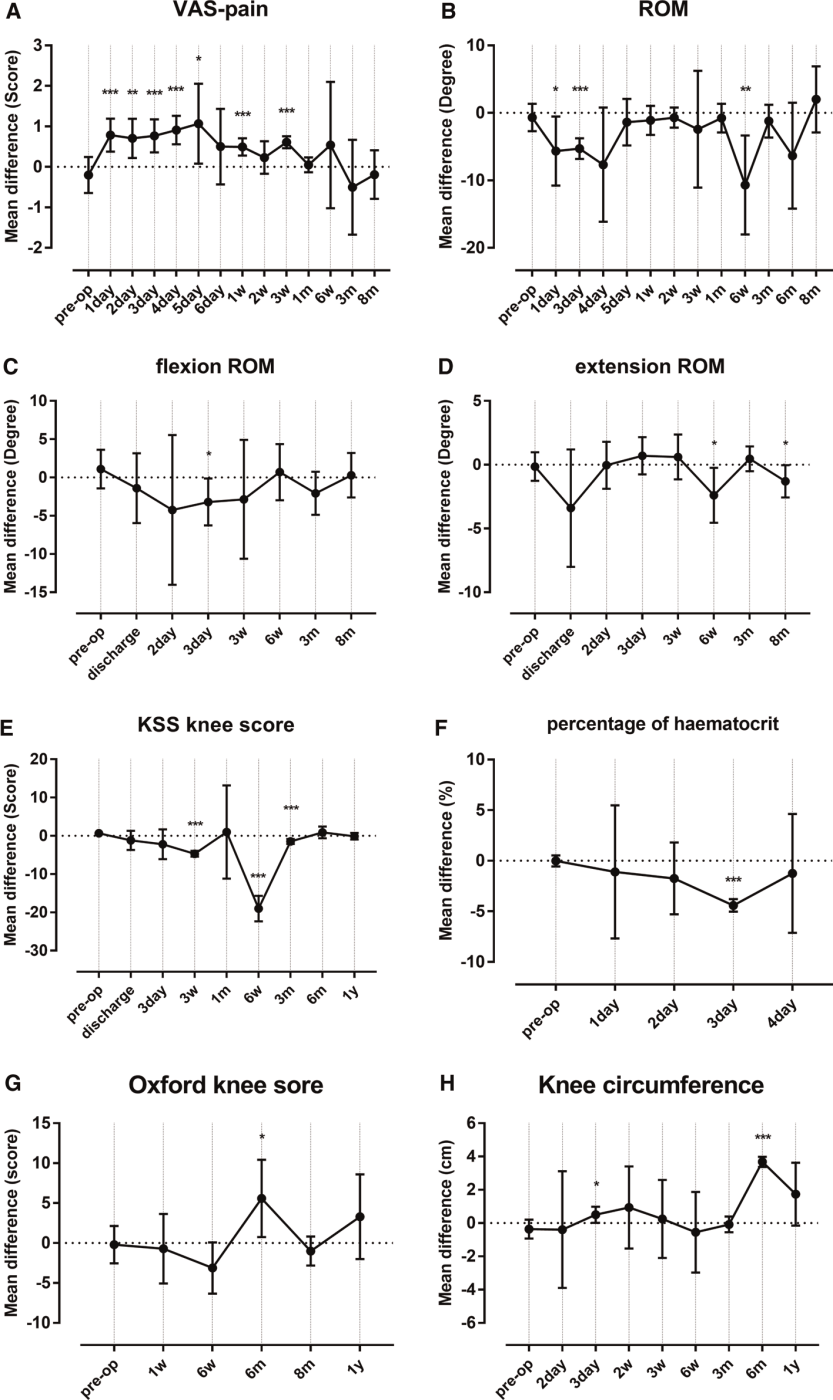
Results for subgroup meta-analyses for outcomes including VAS-pain score (**A**), ROM (**B**), flexion ROM (**C**), extension ROM (**D**), KSS (**E**), percentage of HCT (**F**), Oxford knee score (**G**), and knee circumference (**H**). The error bars in the plot represent mean ± 95%CI. *P < 0.050, **P < 0.010, ***P < 0.001. VAS, visual analog scale; ROM, range of motion; KSS, Knee Society Score; HCT, hematocrit.

Several outcomes were reported at different follow-up points, including VAS-pain score, full ROM, flexion ROM, extension ROM, KSS, percentage of hematocrit (HCT), Oxford knee score, and knee circumference. Thus, subgroup meta-analyses were performed for these outcomes according to the follow-up period (Figure 3). As a result, application of a tourniquet was significantly associated with higher VAS-pain score, at the 1st day, 2nd day, 3rd day, 4th day, 5th day, 1st week, and 3rd week, compared to that of the non-tourniquet group (Figure 3A). Concerning the postoperative ROM, the tourniquet group was associated with significantly decreased full ROM (at the 1st day, 3rd day, and 6th week, Figure 3B), flexion ROM (at the 3rd day, Figure 3C), and extension ROM (at the 6th week and 8th month, Figure 3D). The application of a tourniquet was also related with poorer knee outcome according to KSS at the 3rd week, 6th week, and 3rd month (Figure 3E). The percentage of HCT was significantly lower at the 3rd day postoperatively in the tourniquet group (Figure 3F). The Oxford knee score was higher in the tourniquet group only at the 6th month postoperatively (Figure 3G). Increased swelling was demonstrated for application of tourniquet at the 3rd day and 6th month according to knee circumference (Figure 3H).

**Figure 4:**
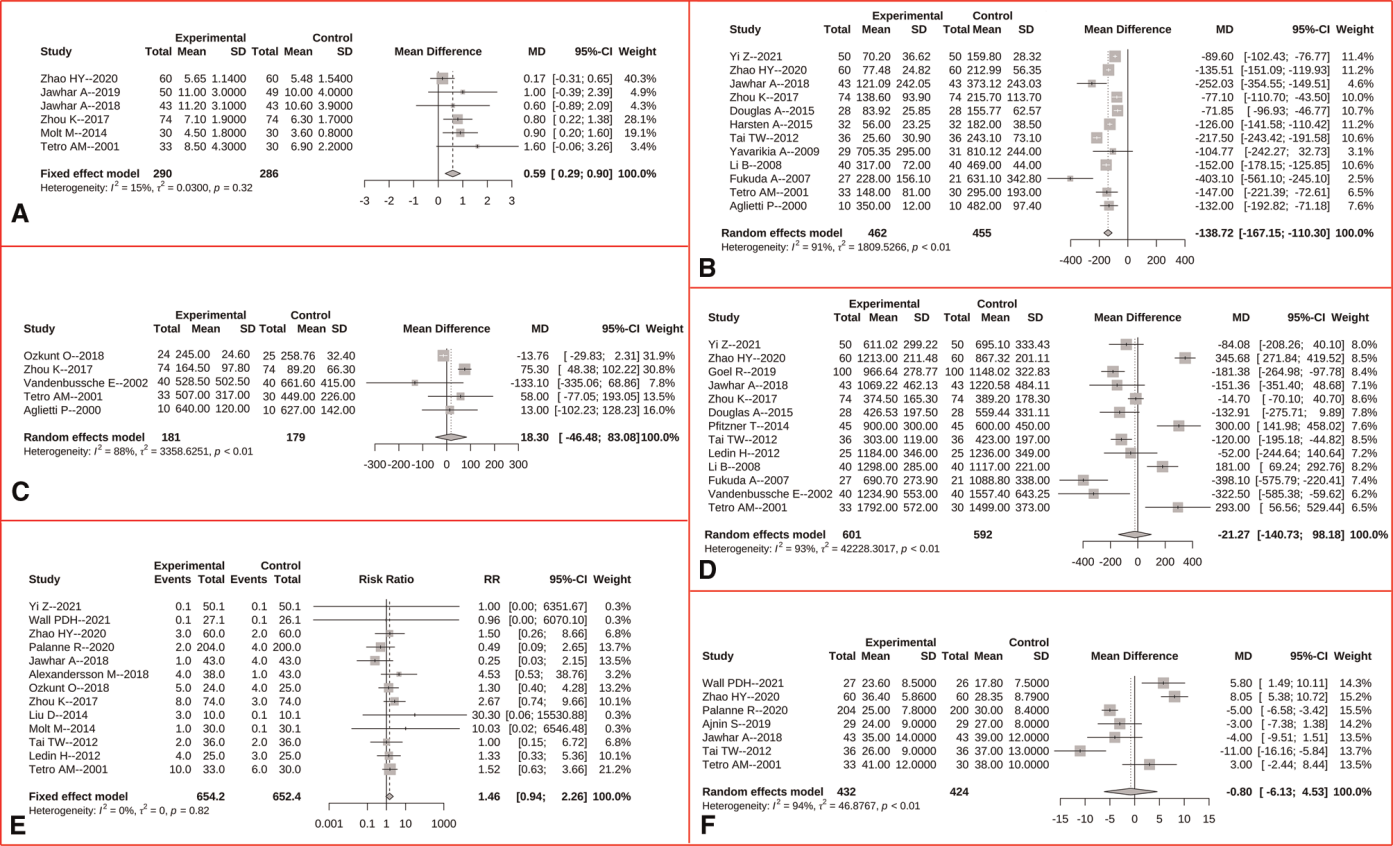
Forest plots for comparisons of length of hospital stay (**A**), intraoperative blood loss (**B**), draining volume (**C**), total blood loss (**D**), transfusion rate (**E**), and hemoglobin change (**F**) between the tourniquet (experimental) and non-tourniquet (control) groups. The *I*^2^ were 15%, 91%, 88%, 93%, 0%, and 94% for the fixed-effects, random-effects, random-effects, random-effects, fixed-effects, and random-effect models, respectively.

The forest plot for comparison of operation time is shown in Supplementary Figure S1. A total of 19 studies were included, presenting that a significantly shorter period was required in TKA with assistance of a tourniquet than TKA without a tourniquet (MD = −1.77 min, 95% CI: −2.61 to −0.93, *p* < 0.001). The forest plot for LOS is presented in Figure 4A, showing increased LOS after application of a tourniquet (MD = 0.59 days, 95% CI: 0.29–0.90, *p* < 0.001).

The intraoperative blood loss was significantly decreased after tourniquet application (MD = −138.72 ml, 95% CI: −167.12 to −110.30, *p* < 0.001, Figure 4B). However, no significant difference was found between two groups for postoperative draining volume (MD = 18.30 ml, 95% CI: −46.48 to 83.08, *p* = 0.580, Figure 4C), total blood loss (MD = −21.27 ml, 95% CI: −140.73 to 98.18, *p* = 0.727, Figure 4D), rate of blood transfusion (RR = 1.46, 95% CI: 0.94–2.26, *p* = 0.095, Figure 4E), and change of the lowest level of Hb compared to baseline level (MD = −0.80 g/L, 95%CI: −6.13 to 4.53, *p* = 0.769, Figure 4F).

**Figure 5:**
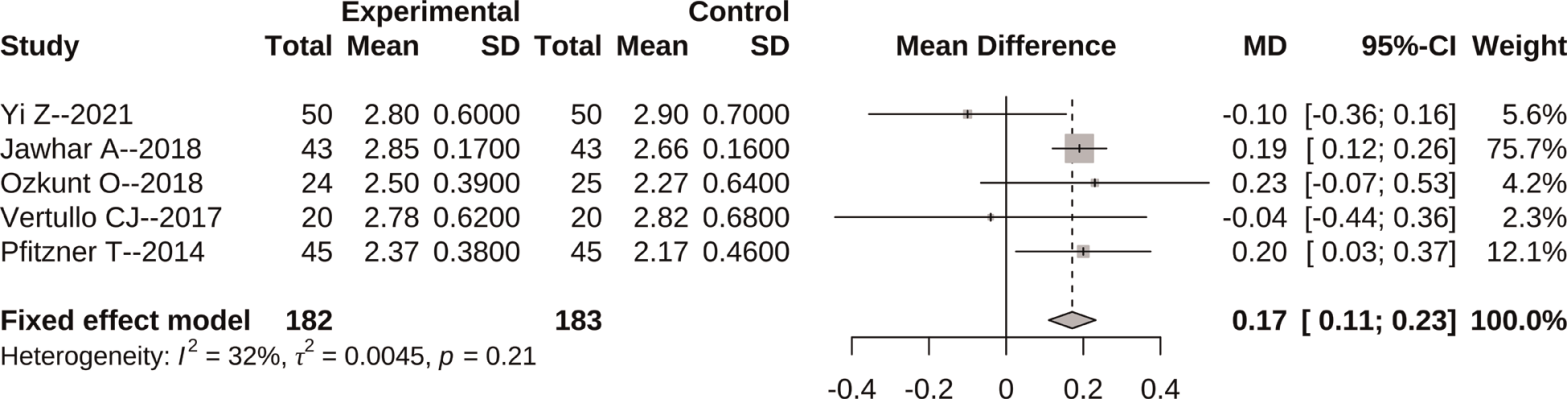
Forest plot for comparison of bone cement mantle thickness between the tourniquet (experimental) and non-tourniquet (control) groups. The *I*^2^ was 32%, and the fixed-effect model was selected.

Data about bone cement mantle thickness were available in five studies, and the corresponding forest plot is presented in Figure 5. As a result, tourniquet application during TKA was related with a significantly increased thickness of bone cement (MD = 0.17 mm, 95% CI: 0.11–0.23, *p* < 0.001, Figure 5).

**Figure 6:**
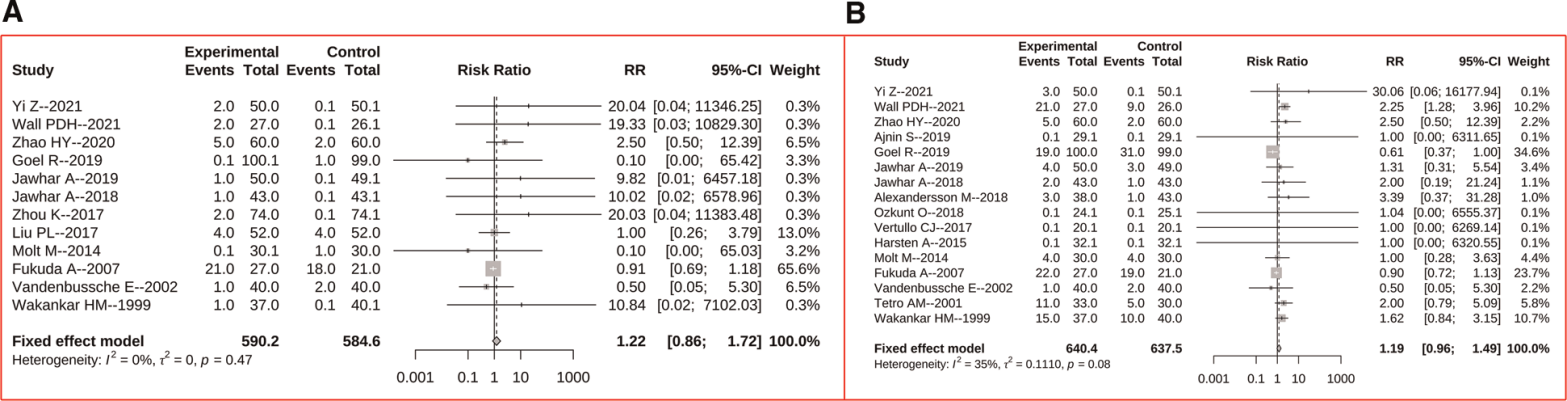
Forest plots for comparisons of risk of DVT (**A**) and risk of all complications (**B**) between the tourniquet (experimental) and non-tourniquet (control) groups. The *I*^2^ were 0% and 35%, and fixed-effect models were selected, respectively.

The risk of DVT was reported in 12 trials, and an overall similar rate of DVT was found between two groups (RR = 1.22, 95% CI: 0.86–1.72, *p* = 0.261, Figure 6A). The frequency of all recorded complications were reported in 16 studies, and no significant difference between two group was demonstrated (RR = 1.19, 95% CI: 0.96–1.49, *p* = 0.118, Figure 6B).

### Sensitivity analyses and publication bias test

When significant heterogeneity was detected in a meta-analysis with more than three studies, sensitivity analysis was performed. As a result, two, one, and three studies were detected to cause significant nonstability to the pooling results, for outcomes of operation time, draining volume, and LOS, respectively (Supplementary Table S1). Thus, these studies were excluded from the final forest plots.

Significant publication bias was found for risk of DVT, according to Egger's test (*p* = 0.088 < 0.100, Supplementary Table S1). The nonparametric trim-and-fill method adjusted the publication bias, and the adjusted effect size was RR = 0.929 (95% CI: 0.720–1.197, *p* = 0.568, *I*^2 ^= 0.0%, fixed-effect model) after adding four studies. The funnel plot of the trim-and-fill process is available in Supplementary Figure S2.

## Discussion

The use of a tourniquet in TKA is controversial, with proponents of tourniquet use claiming possible benefits such as less blood loss, shorter operation time, and thicker cement penetration, while the opponents were concerned about the potential disadvantages of tourniquet application such as increased postoperative pain intensity, increased knee swelling, higher risk of thromboembolic events, and poorer knee function (43). Thus, the exact effectiveness of a tourniquet is not clear now, with conflicting evidence available in recent literature. The main results of the current study include the following: (1) tourniquet application could decrease intraoperative blood loss, shorten operation duration, and increase cement mantle thickness significantly; (2) tourniquet application was significantly associated with increased postoperative pain intensity, decreased full ROM/flexion ROM/extension ROM, poorer knee function, increased knee welling, and increased LOS; (3) no significant difference was found for postoperative draining volume, total blood loss, transfusion rate, change of Hb level, and risks of DVT and all complications.

Extensive blood loss is a major concerning during TKA for orthopedic surgeons, which may cause poor vision during operation and increase the difficulty of operation and risk of injury to some important structures. Thus, various blood-saving techniques have been applied for reducing the intraoperative blood loss. In our study, we confirmed that tourniquet could effectively reduce the intraoperative blood loss, with a mean volume of 138.72 ml, during TKA. Additionally, the operation time was shorter after applying the tourniquet, which may be due to the better intraoperative visualization. It is easy to speculate that tourniquet application is associated with more clear surgical field and can help the surgeons more easily distinguish the anatomical structures, which is great importance for shortening the operation time. However, considering from clinical perspective, the magnitude of the decreased operation time was of no practical significance, as only an average period of 1.77 (95% CI: 0.93–2.61) min was saved. In the meta-analysis of Cai et al. (4), they also showed a similar value of operation time saved in TKA with the assistance of tourniquet (average: 1.08 min, 95% CI: 0.66–1.50 min).

Although many studies have shown decreased intraoperative blood loss with the use of a tourniquet, mixed results have been reported for postoperative and total blood loss. Smith and Hing (12) reported an average of 269 ml increased intraoperative blood loss when no tourniquet was used, while no significant difference was found on the total blood loss and transfusion requirements. Li et al. (44) compared the clinical outcomes of patients who had undergone TKA with or without tourniquet, showing that the tourniquet application was related to decreased intraoperative blood loss, with similar draining volume, total blood loss, and transfusion requirement. In our results, regarding the postoperative draining volume, total blood loss, transfusion rate, and change of Hb level, no significant difference was demonstrated between two groups. On the other hand, some other studies have pointed out that tourniquet application would increase the postoperative hidden blood loss or draining volume (27, 44, 45). Thus, tourniquet application has no influence on the total blood loss and has no effectiveness on reducing transfusion requirement, but only reduces intraoperative bleeding.

We found pain intensity was significantly lower in the non-tourniquet group especially within 3 weeks postoperatively. This may be due to the application of tourniquet that squeezes the thigh and restricts blood flow during operation (7, 46, 47). On the same time, the damage of tourniquet during operation may also cause risk of DVT and knee swelling. This study found a significant increase in knee circumference in the tourniquet group at several follow-ups. However, we did not detect significant difference on frequencies of DVT between the two groups. It could be speculated that the power to detect the difference was insufficient due to low incidences of DVT in both groups. Additionally, an average increase of 0.59 days was calculated for the LOS in the tourniquet group, due to increased pain, delayed rehabilitation, and other reverse effects of the tourniquet. For instance, patients operated with tourniquet assistance were found to be with decreased ROM, and KSS, which could be explained by comparatively more swelling and pain in the postoperative period. This is similar with other previous studies (35, 37, 39), which reported that ROM was reduced in the tourniquet group, even at a period as long as 2 years after TKA.

The bone cement mantle thickness is important for the stability of prostheses following primary TKA (48, 49). Increased thickness of the bone cement has been reported to improve the survival and stability of the prosthesis (50, 51). The cement thickness of 3–4 mm between the tibial component and the trabecular bone is considered to be the best status to avoid osteolysis of the surrounding bone and loosening of the prosthesis (52). Previous studies have presented inconsistent results regarding the effect of tourniquet application on the bone cement thickness (16, 24, 26, 29, 32). Yao et al. (53) evaluated the effect of tourniquet application on cement penetration in primary TKA, and they found that there was no influence on cement penetration; the tourniquet application has no effect on reducing blood loss, easing knee pain and improving knee function. In the results of our study, we found a thicker bone cement mantle in the tourniquet group (mean value: 0.17 mm).

Another strategy selected by many surgeons is to inflate the tourniquet during the cementation process of prosthesis, with the aim of decreasing the tourniquet time (54, 55). In the network meta-analysis published by Cao et al. (54), the efficacy and safety of different tourniquet protocols in TKA were compared, showing that a tourniquet during the entire operation process can effectively reduce blood loss but also may cause many safety problems, including DVTs, wound oozing, delayed healing, and serious wound complications, while tourniquet inflation before osteotomy and then deflation after wound closure can effectively shorten the operation time, reduce perioperative bleeding, and reduce postoperative complications.

This study, however, has some limitations that we need to point out. First, as several outcomes were reported at many follow-up periods, we must perform subgroup meta-analyses according to different follow-ups. As a result, the number of primary trials included in each meta-analysis was quite small. Second, some differences on the operation procedure, such as tourniquet products, tourniquet pressure, and type of prosthesis and cement, existed as presented in Table 2. Thus, we could only calculate an overall effect of size difference between the tourniquet and non-tourniquet groups. These may cause inevitable risk of bias to the results.

## Conclusions

Basing on the available high-quality evidence, the current study demonstrated that tourniquet application could significantly decrease intraoperative blood loss, shorten operation duration, and increase cement mantle thickness. However, tourniquet application was significantly associated with increased postoperative pain intensity, decreased full ROM/flexion ROM/extension ROM, poorer knee function, increased knee welling, and increased LOS. No significant difference was found for postoperative draining volume, total blood loss, transfusion rate, change of Hb level, and risks of DVT and all complications. As a result, we recommend that the tourniquet should not be routinely applied during TKA, or surgeons could inflate the tourniquet only during the cementation process.

## Data Availability

The raw data supporting the conclusions of this article will be made available by the authors, without undue reservation.
