# Supplementary material for: Tourniquet application in primary total knee arthroplasty for osteoarthritis: A systematic review and meta-analysis of randomized controlled trials
Source: Front Surg. 2023 Jan 6;9:994795. doi: 10.3389/fsurg.2022.994795 (PMC9852050; doi:10.3389/fsurg.2022.994795)
Supplement: Supplementary file 1 [file Table1.doc]

| **Supplementary Table S1. Results of the meta-analyses for clinical outcomes at different follow-up times** | | | | | | | | | | |
| --- | --- | --- | --- | --- | --- | --- | --- | --- | --- | --- |
| **Outcomes** | **Included studies** | **Patients (n)** | **I^2^ (%)** | **Q test**  **(p value)** | **Effect model** | **ES (95%CI)** | **Z test (p value)** | **Sensitivity analysis** | **Publication bias** | |
|  |  |  |  |  |  |  |  |  | **Egger’s** | **Begg’s** |
| **operation time (min)** | 19^[9,15,19-23,26,27,29,30,33-35,37-41]^ | 1518 | 31 | 0.100 | F | MD: -1.77[-2.61; -0.93] | <0.001*** | 2^[17,36]^ | 0.389 | 0.753 |
| **intraoperation blood loss (ml)** | 12^[9,15,17,23,26,30,34,36-38,40,41]^ | 917 | 91 | <0.010 | R | MD: -138.72[-167.15; -110.30] | <0.001*** | 0 | 0.250 | 0.273 |
| **draining volume (ml)** | 5^[25,26,39,40,41]^ | 360 | 88 | <0.010 | R | MD: 18.30[-46.48; 83.08] | 0.580 | 1^[15]^ | 0.809 | 1.000 |
| **total blood loss (ml)** | 13^[9,15,17,21,23,26,32,34,35,37-40]^ | 1193 | 93 | <0.010 | R | MD: -21.27 [-140.73; 98.18] | 0.727 | 0 | 0.646 | 0.714 |
| **LOS (day)** | 6^[17,22,23,26,33,40]^ | 576 | 15 | 0.319 | F | MD: 0.59 [0.29; 0.90] | <0.001*** | 3^[15,18,34]^ | 0.181 | 0.348 |
| **bone cement mantle thickness (mm)** | 5^[15,23,25,28,32]^ | 365 | 32 | 0.206 | F | MD: 0.17 [0.11; 0.23] | <0.001*** | 0 | 0.298 | 0.327 |
| **change of Hb between baseline and lowest level (g/L)** | 7^[16-18,20,23,34,40]^ | 856 | 93 | <0.001 | R | MD: -0.80 [-6.13; 4.53] | 0.769 | 0 | 0.641 | 0.652 |
| **transfusion rate (n)** | 13^[15-18,23-26,31,33-35,40]^ | 1306 | 0 | 0.817 | F | RR: 1.46 [0.94; 2.26] | 0.095 | 0 | 0.767 | 0.807 |
| **DVT (n)** | 12^[15-17,21-23,26,27,33,38,39,42]^ | 1174 | 0 | 0.469 | F | RR: 1.22 [0.86; 1.72] | 0.261 | 0 | 0.088* | 0.583 |
| **all complications (n)** | 16^[15-17,20-26,28,30,33,38-40,42]^ | 1425 | 35 | 0.079 | F | RR: 1.19 [0.96; 1.49] | 0.118 | 0 | 0.138 | 0.589 |
| **VAS-pain score** | | | | | | | | | | |
| pre-op | 9^[9,16,19,21,23,24,26,27,32]^ | 888 | 77 | <0.010 | R | MD: -0.20 [-0.65; 0.24] | 0.375 | 0 | 0.846 | 0.532 |
| 1day | 9^[15-18,20,27,29,31,34]^ | 986 | 84 | <0.010 | R | MD: 0.79 [0.38; 1.19] | <0.001*** | 0 | 0.584 | 0.677 |
| 2day | 8^[9,16,17,20,29,31,32,34]^ | 528 | 73 | <0.010 | R | MD: 0.71 [0.22; 1.19] | 0.004** | 0 | 0.685 | 0.621 |
| 3day | 8^[15,17,24,26,27,29,31,34]^ | 705 | 89 | <0.010 | R | MD: 0.77 [0.36; 1.17] | <0.001*** | 0 | 0.748 | 0.621 |
| 4day | 4^[17,31,32,34]^ | 302 | 50 | 0.110 | R | MD: 0.91 [0.56; 1.26] | <0.001*** | 0 | - | - |
| 5day | 3^[26,27,31]^ | 272 | 96 | <0.010 | R | MD: 1.07 [0.08; 2.05] | 0.034* | - | - | - |
| 6day | 1^[23]^ | 86 | - | - | F | MD: 0.50 [-0.43; 1.43] | 0.293 | - | - | - |
| 1w | 2^[16,27]^ | 155 | 0 | 0.640 | F | MD: 0.49 [0.28; 0.71] | <0.001*** | - | - | - |
| 2w | 2^[27,29]^ | 164 | 83 | 0.020 | R | MD: 0.23 [-0.17; 0.63] | 0.261 | - | - | - |
| 3w | 2^[9,26]^ | 204 | 0 | 0.810 | F | MD: 0.61 [0.46; 0.76] | <0.001*** | - | - | - |
| 1m | 2^[19,27]^ | 174 | 0 | 0.820 | F | MD: 0.05 [-0.13; 0.23] | 0.589 | - | - | - |
| 6w | 3^[21,25,29]^ | 309 | 99 | <0.001 | R | MD: 0.54 [-1.02; 2.10] | 0.498 | - | - | - |
| 3m | 3^[9,19,24]^ | 207 | 81 | <0.010 | R | MD: -0.50 [-1.67; 0.67] | 0.402 | - | - | - |
| 8m | 1^[21]^ | 200 | - | - | F | MD: -0.19 [-0.79; 0.41] | 0.535 | - | - | - |
| **full ROM** | | | | | | | | | | |
| pre-op | 6^[17,19,26,27,35,37]^ | 572 | 0 | 0.759 | F | MD: -0.68 [-2.70; 1.34] | 0.507 | 0 | 0.846 | 0.573 |
| 1day | 1^[26]^ | 148 | - | - | F | MD: -5.65 [-10.77; -0.53] | 0.030* | - | - | - |
| 3day | 4^[15,17,26,35]^ | 418 | 19 | 0.296 | F | MD: -5.29 [-6.83; -3.76] | <0.001*** | 0 | - | - |
| 4day | 1^[35]^ | 50 | - | - | F | MD: -7.67 [-16.12; 0.78] | 0.075 | - | - | - |
| 5day | 1^[26]^ | 148 | - | - | F | MD: -1.37 [-4.81; 2.07] | 0.436 | - | - | - |
| 1w | 1^[9]^ | 104 | - | - | F | MD: -1.10 [-3.25; 1.05] | 0.317 | - | - | - |
| 2w | 1^[9]^ | 104 | - | - | F | MD: -0.70 [-2.18; 0.78] | 0.354 | - | - | - |
| 3w | 2^[17,26]^ | 268 | 92 | <0.001 | R | MD: -2.42 [-11.08; 6.24] | 0.584 | - | - | - |
| 1m | 2^[19,27]^ | 174 | 8 | 0.299 | F | MD: -0.77 [-2.90; 1.36] | 0.477 | - | - | - |
| 6w | 1^[35]^ | 50 | - | - | F | MD: -10.68 [-18.00; -3.36] | 0.004** | - | - | - |
| 3m | 6^[15,17,19,26,27,35]^ | 592 | 68 | 0.009 | R | MD: -1.21 [-3.64; 1.22] | 0.328 | 0 | 0.606 | 0.573 |
| 6m | 3^[20,26,35]^ | 256 | 94 | <0.001 | R | MD: -6.34 [-14.19; 1.52] | 0.114 | - | - | - |
| 8m | 1^[20]^ | 58 | - | - | F | MD: 2.00 [-2.90; 6.90] | 0.423 | - | - | - |
| **flexion ROM** | | | | | | | | | | |
| pre-op | 3^[9,21,24]^ | 337 | 34 | 0.219 | F | MD: 1.10 [-1.43; 3.63] | 0.393 | - | - | - |
| discharge | 1^[21]^ | 200 | - | - | F | MD: -1.40 [-5.96; 3.16] | 0.548 | - | - | - |
| 2day | 1^[9]^ | 104 | - | - | F | MD: -4.24 [-14.02; 5.54] | 0.396 | - | - | - |
| 3day | 1^[24]^ | 81 | - | - | F | MD: -3.20 [-6.26; -0.14] | 0.041* | **-** | **-** | **-** |
| 3w | 1^[9]^ | 104 | - | - | F | MD: -2.85 [-10.62; 4.92] | 0.472 | - | - | - |
| 6w | 1^[21]^ | 200 | - | - | F | MD: 0.70 [-2.97; 4.37] | 0.709 | **-** | **-** | **-** |
| 3m | 2^[9,24]^ | 137 | 0 | 0.864 | F | MD: -2.06 [-4.87; 0.74] | 0.149 | **-** | **-** | **-** |
| 8m | 1^[21]^ | 200 | - | - | F | MD: 0.30 [-2.60; 3.20] | 0.839 | **-** | **-** | **-** |
| **extension ROM** | | | | | | | | | | |
| pre-op | 3^[9,21,24]^ | 337 | 0 | 0.998 | F | MD: -0.14 [-1.26; 0.98] | 0.807 | - | - | - |
| discharge | 1^[21]^ | 200 | - | - | F | MD: -3.40 [-8.00; 1.20] | 0.148 | - | - | - |
| 2day | 1^[9]^ | 104 | - | - | F | MD: -0.04 [-1.88; 1.80] | 0.966 | - | - | - |
| 3day | 1^[24]^ | 81 | - | - | F | MD: 0.70 [-0.75; 2.15] | 0.345 | - | - | - |
| 3w | 1^[9]^ | 104 | - | - | F | MD: 0.61 [-1.15; 2.37] | 0.497 | - | - | - |
| 6w | 1^[21]^ | 200 | - | - | F | MD: -2.40 [-4.55; -0.25] | 0.029* | - | - | - |
| 3m | 2^[9,24]^ | 137 | 0 | 0.822 | F | MD: 0.47 [-0.50; 1.43] | 0.345 | - | - | - |
| 8m | 1^[21]^ | 200 | - | - | F | MD: -1.30 [-2.56; -0.04] | 0.044 | - | - | - |
| **KSS knee score** | | | | | | | | | | |
| pre-op | 6^[15,17,19,25-27]^ | 591 | 0 | 0.737 | F | MD: 0.68 [-0.34; 1.71] | 0.191 | 0 | 0.797 | 0.851 |
| discharge | 1^[26]^ | 148 | - | - | F | MD: -1.20 [-3.70; 1.30] | 0.347 | - | - | - |
| 3day | 2^[15,17]^ | 220 | 96 | <0.001 | R | MD: -2.19 [-6.07; 1.69] | 0.268 | - | - | - |
| 3w | 1^[17]^ | 120 | - | - | F | MD: -4.68 [-5.45; -3.91] | <0.001*** | - | - | - |
| 1m | 1^[19]^ | 70 | - | - | F | MD: 1.00 [-11.18; 13.18] | 0.872 | - | - | - |
| 6w | 1^[25]^ | 49 | - | - | F | MD: -19.00 [-22.33; -15.67] | <0.001*** | - | - | - |
| 3m | 4^[17,19,26,27]^ | 442 | 38 | 0.201 | F | MD: -1.48 [-2.13; -0.82] | <0.001*** | - | - | - |
| 6m | 1^[26]^ | 148 | - | - | F | MD: 0.90 [-0.62; 2.42] | 0.245 | - | - | - |
| 1y | 1^[27]^ | 104 | - | - | F | MD: -0.10 [-0.97; 0.77] | 0.821 | - | - | - |
| **percentage of HCT (%)** | | | | | | | | | | |
| pre-op | 3^[17,34,37]^ | 272 | 0 | 0.387 | F | MD: -0.01 [-0.56; 0.53] | 0.958 | - | - | - |
| 1day | 2^[17,34]^ | 192 | 97 | <0.001 | R | MD: -1.11 [-7.68; 5.46] | 0.741 | - | - | - |
| 2day | 3^[17,20,34]^ | 250 | 94 | <0.001 | R | MD: -1.74 [-5.28; 1.79] | 0.335 | - | - | - |
| 3day | 1^[17]^ | 120 | - | - | F | MD: -4.41 [-5.03; -3.79] | <0.001*** | - | - | - |
| 4day | 2^[17,34]^ | 192 | 97 | <0.001 | R | MD: -1.24 [-7.11; 4.63] | 0.678 | - | - | - |
| **Oxford Knee Score** | | | | | | | | | | |
| pre-op | 2^[16,22]^ | 151 | 19 | 0.266 | F | MD: -0.20 [-2.54; 2.14] | 0.869 | - | - | - |
| 1w | 1^[16]^ | 47 | - | - | F | MD: -0.70 [-5.06; 3.66] | 0.753 | - | - | - |
| 6w | 3^[16,20,22]^ | 209 | 70 | 0.036 | R | MD: -3.12 [-6.32; 0.09] | 0.056 | - | - | - |
| 6m | 1^[16]^ | 47 | - | - | F | MD: 5.60 [0.76; 10.44] | 0.023* | - | - | - |
| 8m | 1^[20]^ | 58 | - | - | F | MD: -1.00 [-2.82; 0.82] | 0.282 | - | - | - |
| 1y | 1^[16]^ | 47 | - | - | F | MD: 3.30 [-2.00; 8.60] | 0.222 | - | - | - |
| **knee circumference (cm)** | | | | | | | | | | |
| pre-op | 4^[9,24,31,37]^ | 237 | 0 | 0.723 | F | MD: -0.36 [-0.93; 0.21] | 0.216 | 0 | - | - |
| 2day | 1^[9]^ | 104 | - | - | F | MD: -0.39 [-3.89; 3.11] | 0.827 | - | - | - |
| 3day | 1^[24]^ | 81 | - | - | F | MD: 0.50 [0.02; 0.98] | 0.043* | - | - | - |
| 2w | 1^[31]^ | 20 | - | - | F | MD: 0.94 [-1.53; 3.41] | 0.456 | - | - | - |
| 3w | 1^[9]^ | 104 | - | - | F | MD: 0.25 [-2.09; 2.59] | 0.835 | - | - | - |
| 6w | 1^[31]^ | 20 | - | - | F | MD: -0.55 [-2.97; 1.87] | 0.657 | - | - | - |
| 3m | 2^[9,24]^ | 137 | 0 | 0.687 | F | MD: -0.08 [-0.55; 0.39] | 0.743 | - | - | - |
| 6m | 1^[31]^ | 20 | - | - | F | MD: 3.68 [3.37; 3.99] | <0.001*** | - | - | - |
| 1y | 1^[31]^ | 20 | - | - | F | MD: 1.74 [-0.15; 3.63] | 0.071 | - | - | - |

Footnote: abbreviations: ES, effect size; LOS, length of hospital stay; MD, mean difference; RR, risk ratio; HCT, haematocrit; F, fixed-effect model; R, random-effect model; ROM, range of motion; DVT, deep vein thrombosis. *p<0.050, **p<0.010, ***p<0.001.
