# Supplementary figures and images for: Tourniquet application in primary total knee arthroplasty for osteoarthritis: A systematic review and meta-analysis of randomized controlled trials
Source: Front Surg. 2023 Jan 6;9:994795. doi: 10.3389/fsurg.2022.994795 (PMC9852050; doi:10.3389/fsurg.2022.994795)

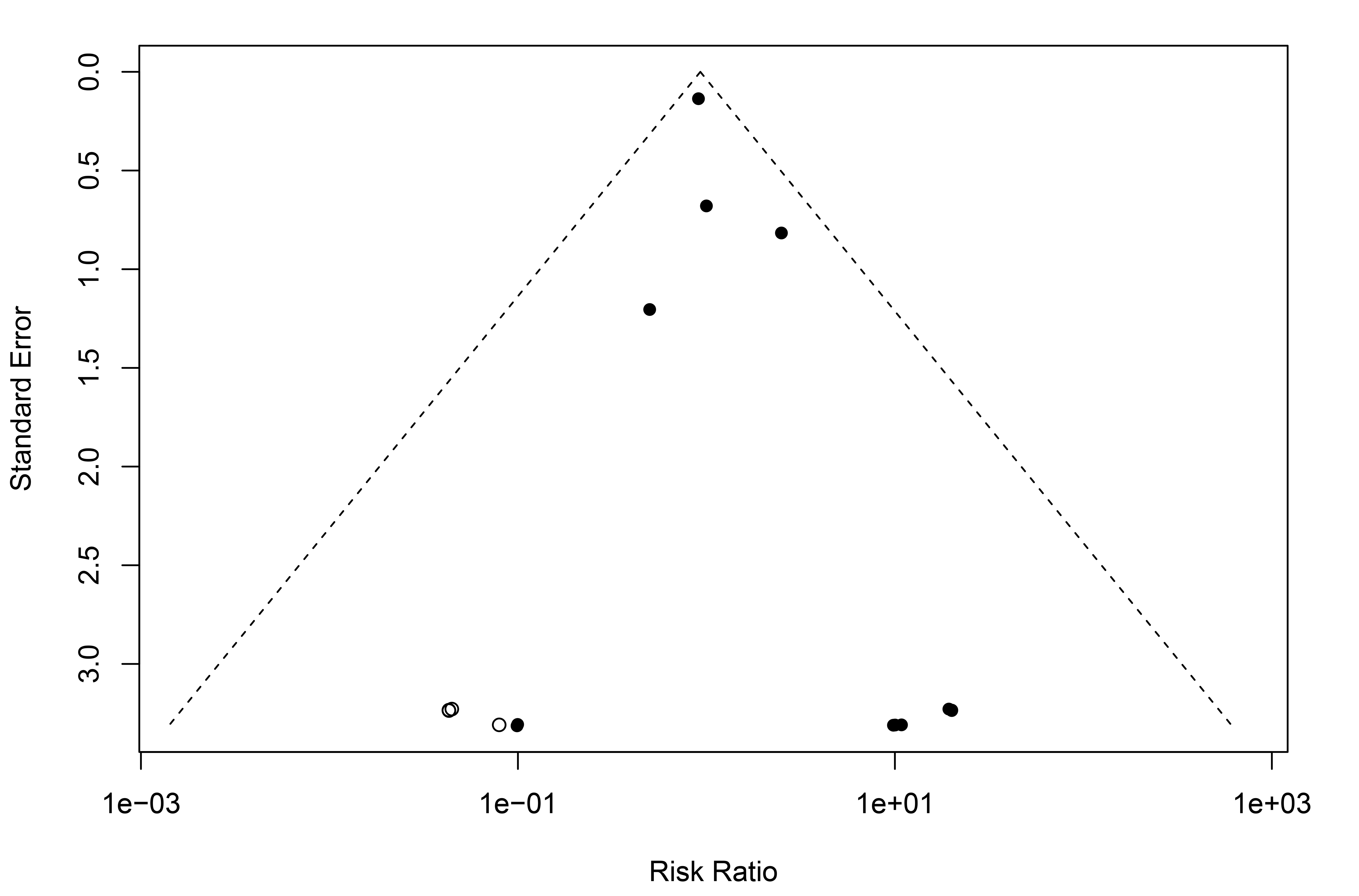

Supplement: Supplementary file 2 [file Image1.tif]

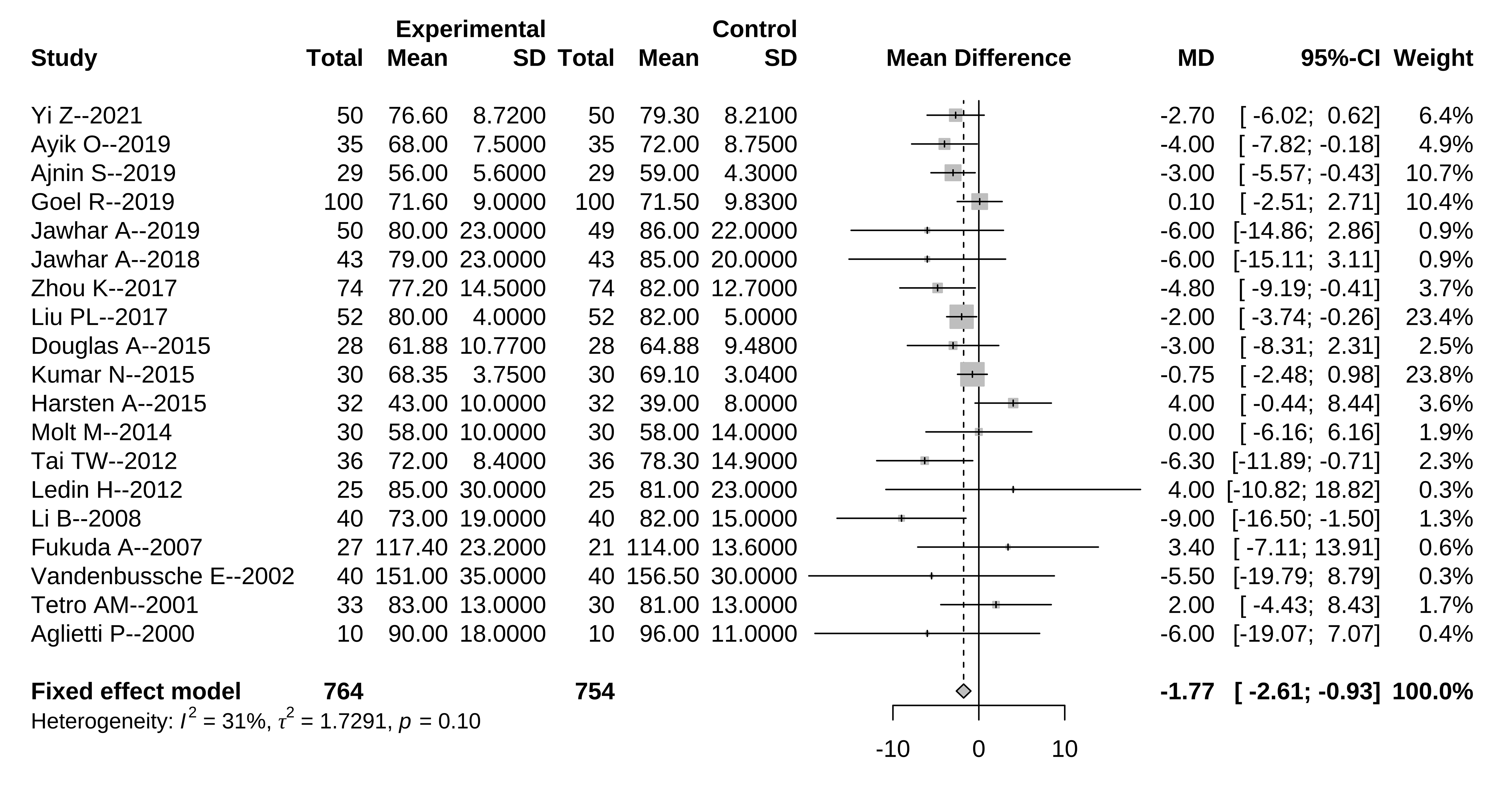

Supplement: Supplementary file 3 [file Image2.tif]
